# The Demography and Practice of Australians Caring for Native Wildlife and the Psychological, Physical and Financial Effects of Rescue, Rehabilitation and Release of Wildlife on the Welfare of Carers

**DOI:** 10.3390/ani9121127

**Published:** 2019-12-12

**Authors:** Bruce Englefield, Steve Candy, Melissa Starling, Paul McGreevy

**Affiliations:** 1 School of Veterinary Science, The University of Sydney, Sydney, NSW 2006, Australia; mjstarling@fastmail.com.au (M.S.); paul.mcgreevy@sydney.edu.au (P.M.); 2 Scandy Statistical Modelling Pty Ltd., 70 Burwood Drive, Blackmans Bay, Tasmania 7052, Australia; burwood70@gmail.com

**Keywords:** wildlife carers, One Welfare, Australian native wildlife, burnout, compassion fatigue, rehabilitation, grief, roadkill rescue

## Abstract

**Simple Summary:**

Little is known about the physical, financial and emotional effects on the 20,000 volunteers who rescue, rehabilitate and release injured and/or orphaned Australian wildlife. A survey for wildlife carers was instigated to address this knowledge gap. Collated survey responses from the wildlife carers suggested that their physical workload is on average 32 h per week but can be up to 100 h, their financial input is on average AUD5300 per year and over a lifetime of caring can go up to AUD800,000. The emotional input is such that 28% of the respondents were experiencing moderate to severe grief. Grief increases as more joeys die in care and as expenditure rises, and is age dependent. Burnout and compassion fatigue are likely outcomes. Over 65% of respondents felt that their welfare, and that of the animals for whom they care, is neglected and unappreciated by government agencies. Unless these deficiencies are corrected by financial and emotional support and workload is reduced for carers, animal and wildlife carer welfare will be compromised.

**Abstract:**

The rescue, rehabilitation and release of injured and orphaned Australian wildlife is managed by over 20,000 carers, mostly voluntarily. These volunteers experience mental, physical and financial challenges that have not been researched adequately. This study collated the responses (*n* = 316) to a survey conducted among Australian wildlife carers who actively foster orphaned joeys for hand-raising and injured adult mammals for rehabilitation and release. It confirmed 86% of rehabilitators are female, 70% are over the age of 46 years and their prime motivation is an affinity with animals. The average time spent in the sector is 11.5 years, and the work week is 31.6 h, caring for 15 animals per year, with an average of 2.6 dying. The average financial commitment is AUD5300 annually and up to AUD800,000 over a lifetime. Regarding the grief experienced by carers, the lower the age, the longer the time spent, the greater the financial input and the more joeys that died, the more severe is the grief experienced. Moderate to severe grief is experienced by 28% of carers, which, coupled with other factors, could lead to burnout or compassion fatigue. Soon, wildlife carer welfare will likely be compromised unless financial and mental support is provided and their workload reduced.

## 1. Introduction

The first law to prevent cruelty to animals in Australia was passed in Van Diemen’s Land (now Tasmania) in 1837 by Governor Sir John Franklin. It was most probably based on British legislation enacted in 1835 (known as Pease’s Act) [1]. By the 1870s, there were four animal protection societies in Australia and, over the next 80 years, the animal protection movement developed to rescue and rehabilitate injured and orphaned wildlife [2]. Currently, wildlife rescue and rehabilitation in Australia can be regarded as a major industry, with over 20,000 employees, albeit mostly volunteers, approximately 17,500 of whom are registered with State or Territory authorities [2,3]. Unfortunately, with no national political leadership or initiative to oversee this operation, it is a fragmented system that is managed on a state or territory basis. This fragmentation has resulted in considerable differences in the legislative and policy framework [4].

Any operation that relies on self-funding and the goodwill of volunteers is vulnerable to internal threats. In this instance, examples of such internal threats include financial pressures, inefficient management, unreliability of workforce numbers, as well as the widespread lack of operational health and safety monitoring of the workforce, scientific and practical protocols for wildlife rehabilitation and monitoring outcomes. External threats involve an increasing number of animals being presented for rescue and rehabilitation [5] and a decrease in the recruitment of new wildlife carers [6]. The increase in rescues reflects several anthropogenic factors, including habitat destruction, predation by introduced species and domestic pets, wildlife–vehicle collisions (WVC), farming practices and culling/hunting. Climate change increases the risk of bushfires, floods and hazardous weather events that influence the need for animal rescue. Habitat destruction has a major effect on the ecosystem, with inestimable numbers of mammals being displaced [7] and requiring rescue.

The threats from WVC and predation are exacerbated by the increasing number of vehicles on Australian roads and pet ownership. The 2015 Motor Vehicle Census reveals over 18 million registered motor vehicles in Australia, a total that had increased to 19.2 million by 2018 [8]. In 1994, in Australia, there were 3.1 million owned dogs (*Canis lupus familiaris*) and 2.5 million owned cats (*Felis catus*) [9]. By 2016, these numbers had increased to 4.8 million dogs and 3.9 million cats [10]. The role that the human‒animal interaction and companion animals play in creating the need for wildlife rescue is confirmed by New South Wales’s Wildlife Information Rescue and Education Service (WIRES), the largest Australian wildlife rehabilitation network, with 28 branches and over 2500 volunteers [11]. The service reported a 19% increase in the number of calls about injured animals in the previous year (2016–2017). For a twelve-month period in 2013–2014, RSPCA Queensland reported that around 8500 native animals and birds were presented at its Wacol wildlife hospital. By 2017–2018, that figure had swollen to well over 23,000.

Predation by introduced species such as the red fox (*Vulpes vulpe*s), feral and domestic cats and, to a lesser extent, domestic dogs, is a major threat [12]. The animals that, as a result, are injured or orphaned and rescued then face additional challenges in successful reintroduction. Wildlife carers are mandated to return the animals to the location from which they were rescued; so after rehabilitation, the continued presence of these predators in their habitat reduces the rescued animals’ chances of survival [4].

Many injured and orphaned wildlife require euthanasia, but those assessed as suitable for rehabilitation require wildlife carers to take total responsibility for them. The duration of full rehabilitation and release can be lengthy, e.g., up to two years for some hand-reared marsupial neonates (joeys). What the animals learn during this time can be critical to the success of their eventual release. An animal that has been humanized, i.e., desensitized to sights, sounds and odors associated with humans, is unlikely to survive in the wild or have a life worth living, as it is likely to experience anxiety, fear, panic, frustration, anger, helplessness, loneliness, boredom and depression [13]. Codes of practice recognize this and state that ‘releasing wildlife is the most difficult part of the rehabilitation process but should be considered the most important’ and ‘release options and procedures should be of the highest priority and taken into consideration at the time of acquisition of any wildlife’ [14]. However, in 2000 [15], 2005 [16] and 2010 [7], it was reported that scientific studies on the pre-release treatment of animals in animal welfare-based rehabilitation of Australian mammals simply did not exist. Eight years later they still do not exist. The lack of pre- and post-release, science-based protocols for rescued wildlife monitoring presents a worrying gap in knowledge.

Another major threat to the wildlife carer network is the finite number of trained volunteers to meet increasing demand. A recent report on wildlife volunteers in New South Wales highlighted ‘some groups reporting fluctuations in annual membership of 25% and in some areas 60%’ [2]. Although wildlife carers may be highly motivated volunteers, they require the same attention to occupational health and safety as commercial operators would be required to undertake in a well-functioning and reliable workforce.

Over the past 20 years, codes of practice designed to benefit the wellbeing of wildlife have been established in all Australian states to address wildlife carer training, regulation and the general structure of wildlife rehabilitation. However, two significant and persistent gaps in understanding and addressing the wellbeing of wildlife carers related to compassion fatigue (CF), described as ‘a state of exhaustion and dysfunction—biologically, psychologically, and socially—as a result of prolonged exposure to compassion stress and all that it evokes’ [17], and to burnout, defined as a ‘state of emotional, physical, and mental exhaustion caused by excessive and prolonged stress. … [occurring] … when you feel overwhelmed, emotionally drained, and unable to meet constant demands’ [18,19]. Both CF and burnout are recognized in other healthcare professionals such as intensive care unit nurses and doctors [20], military medical staff [21], audiologists [22] and mental health practitioners [23], but the possibility of their occurrence in the wildlife rehabilitation sector has not been explored in Australia. A recent survey of wildlife carers in New Zealand [24] concluded that there were significant differences in CF among New Zealand wildlife carers based on their age, gender, financial capacity and years of experience but that, overall, the incidence of CF was considered low, at an estimated 20%. However, the sample size of wildlife carers in that study, undertaken with attendees at the Wildlife Rehabilitators Network of New Zealand Conference held at Massey University in 2016, was small (*n* = 30), so the results should be viewed with caution. It is possible that the estimated CF percentage represents an under-estimate, especially if wildlife carers with the time, financial means and motivation to attend such a conference are not those likely to be suffering burnout or CF.

An international survey of wildlife carers (*n* = 534) was conducted to collect respondents’ thoughts and feelings on mental health awareness [25]. Bullying, isolation, grief and financial burden were the four most significant themes explored and were proposed as reasons why carers might leave organizations or give up caring altogether. It concluded that deeper research is required to ensure that the impacts of positive and negative mental health consequences on wildlife carers are better understood.

The current study is the first to encompass wildlife carers from all states and territories of Australia. It involved a survey that was open to all Australian adults who rescued, rehabilitated and released marsupial mammals. The focus on mammals was implemented to target on the areas where hand-rearing, time in captivity, financial resources, animal behavior modification and wellbeing of carers combine to be of major significance. The aim was to assess what motivates people to become wildlife carers, their demographics, the number of animals and the species they rehabilitate, their opinion on the release methodology they use, release protocols, how well they feel supported in their role as wildlife carers, their overall financial and time contribution and the effect that wildlife rescue and rehabilitation has on their mental wellbeing as measured by the amount of grief experienced. The combined effect of these factors, coupled with lack of recognition or support and work overload, could indicate how exposed the wildlife carers are to CF or burnout.

Our overarching aim was to understand the complexities and size of the wildlife rescue and rehabilitation operation. The results were expected to reveal the threats to the future of such operations and to identify areas for potential improvement that could optimize the wellbeing of native animals and wildlife carers.

## 2. Materials and Methods

A quantitative and qualitative questionnaire was developed and set up online using the Research Electronic Data Capture (REDCap) system developed by a multi-institutional consortium initiated at Vanderbilt University USA in 2017. Data collection was customized for the current study by the research team with guidance from support staff at the University of Sydney. Ethics approval was obtained from the Human Research Ethics Committee at the University of Sydney under approval number: 2017/492.

The questionnaire was open to all adult Australian wildlife carers who rescued, rehabilitated and released marsupial mammals. A hyperlink containing a participant information statement, information about the project and the survey questionnaire was sent by email to wildlife carer networks across Australia inviting them to distribute it to appropriate contacts. It was also sent to individual wildlife carer license holders registered on websites. It was proposed that wildlife carers who did not have access to the internet could be invited by friends or colleagues to use their facilities to complete the questionnaire online. It was explained that all replies would be anonymous so that an individual’s information would not be identifiable. However, respondents had the option to submit their email address via a separate email to the project supervisor if they wished to receive feedback on the survey. Participation in the survey was voluntary and participants could withdraw from the survey at any point by not submitting a response. Conversely, participation implied consent to the research.

The survey had four sections: demographics and motivations; contributions; knowledge and experience and mental health. Questions in the first three sections were mandatory, whereas the mental health section was optional. The questions on motivation were formulated using studies on the motivations of general volunteers and volunteers in wildlife rehabilitation [26,27]. A grief diagnostic instrument was used to evaluate the grief experienced by wildlife carers [28]. The category scores in the mental health section were based on the Diagnostic and Statistical Manual of Mental Disorders (DSM-1V) of the American Psychiatric Association [29] by the authors of the General Diagnostic Instrument [28]. A free-text general question invited the respondents to add any comments that they felt might clarify the role carers play in rescuing, rehabilitating and releasing native marsupial wildlife. If the respondents declined to answer the mental health section, they were directed to the final question, asking them if they wished to receive feedback on the results of the survey.

The questionnaire collected information on respondents’ gender, age, ethnicity, birthplace, first language and place of abode. There were 13 options on what motivated the participants to become wildlife carers, and participants were asked to select as many as applied to them. The options were: I had an affinity with animals and a desire to help them; to help conserve the environment; to contribute to my community; I had the skills to rear animals; I related better with animals than humans; to learn something new; to handle animals; to challenge myself; I believed that I had a special gift in relating to animals; to socialize and meet people; to do something different; to fill a void in my life and to help me get a future job working with animals (see Table 4). Note that respondents could nominate more than one category of motivation, so the proportions across categories were not independent. Therefore, we used Markov Chain Monte Carlo (MCMC) sampling to model the set of multivariate binomial responses (i.e., response of “yes” or “no” for each of the 13 categories of motivation) using the MCMCglmm function in the MCMCglmm R-software library [30]. The null hypothesis that the proportion of males and females identifying a motivation category as a “yes” was the same, was tested by the male parameter estimate for each motivation category and determining that the MCMC 95% support interval did not include zero (i.e., reject null hypothesis at the *p* < 0.05 probability level).

Respondents were asked to report the species of animals that they had hand-reared, number of each species, the number of animals that died whilst in care and the financial and time input devoted to caring for the animals. They were also asked to give an approximation of the average hours per week they had spent doing volunteer work as a wildlife carer, during the year 1st July 2016 to 30th June 2017.

There were 16 questions on the length of service of wildlife carers, formal instruction or training received, methods used for the pre- and post-release of animals, identification of animals, and respondents’ opinion on housing, release methodology and improvements that could be made to optimize welfare for the animals and the financial and time input of carers (Table 1).

Respondents could select one of five methods that they used when releasing rehabilitated animals. Respondents were also asked to mention the methods of release they would prefer to use if given a choice; i.e., one not constrained by regulations or codes of practice. Respondents were asked to select the place where they would prefer to house a rehabilitated animal if it required temporary housing prior to release and about the fate of an animal if it was deemed unsuitable for release into the wild after rehabilitation.

Three questions explored how well wildlife carers thought they were supported in their role. Nine further questions concerned distressing events, regarded as losses, that may have occurred in the last year and participants were asked to select those they had experienced (Table 2).

A validated grief diagnostic instrument (GDI) [28] was used to assess the grief experienced by wildlife carers (Table 3).

Participants were asked to score on a Likert scale of 1–4 with the options: a lot of the time; quite a bit of the time; a little bit of the time and never.

Several methods were used to undertake statistical analyses. Cronbach’s alpha was used to measure the reliability of the GDI diagnostic instrument. To investigate potential risk factors that predict GDI scores, five predictor variables were considered; age of carer at the time of the survey, years of experience as a carer, number of joeys that died in their care in the previous year (2016–2017), average total hours per week spent caring in the previous year and the Napierian logarithmic transform of total financial cost of caring in previous year. All erroneously recorded values such as zero total hours spent and zero costs and total hours per week that were unrealistically high (i.e., greater than 100 hrs per week) were excluded. After this processing, data for 207 participants were useable for modeling. The logarithmic transform of total financial cost of caring in previous year was required to avoid excessive leverage of this variable on the GAM fit because of its extreme range in values (i.e., range of AUD150 to AUD89,000).

The age of carer categories was fitted in a Generalised Additive Model (GAM) [31] as a factor, while the other four continuous variables were each fitted as cubic smoothing spline terms with a basis dimension of five in each case. These five predictor variables were fitted jointly to GDI score, assuming Gaussian errors for the scores combined with the identity link function. Residual plots showed that the residuals from the fit had positive skew. However, transforming the scores using the square root function and refitting the GAM to this transformed response variable removed the skew in residuals and gave very close to Gaussian-distributed residuals as verified using a quantile–quantile (qq) plot. The predictor variables were combined additively in the GAM and interactions between them (such as fitting the smooths separately to each age category) were not investigated due to insufficient data. Combinations of the predictor variables were fitted, and the best model was selected based on its Akaike Information Criterion (AIC), which combines the lack of fit (i.e., minus twice the log-likelihood) with a penalty for number of parameters fitted. To demonstrate the effect of these variables on GDI score, for each variable in turn, the predicted mean GDI score (on the square root scale) was graphed against that variable while the other two variables were fixed at a single value.

When comparing the financial input and time spent in caring the fitted locally estimated scatterplot smoothing (LOESS) smooth (solid line) and the gamma generalized linear model (GLM) fit, used the identity link function (i.e., a linear model) with intercept constrained to zero. The comparison of the LOESS and GLM fit was close, so that a linear relationship was justified.

## 3. Results

Of the 316 responses received, 270 were complete. This represented 7.6% of the approximately 4150 wildlife carers in Australia eligible and able to access the survey, i.e., those caring for marsupials in the year 2016–2017 and with internet access. Optional questions, designed to measure general grief, were declined by 36 participants. A further ten participants failed to complete one or more questions in the other section of the questionnaire. This meant that data from a total of 270 respondents were available for full analysis.

**Figure 1:**
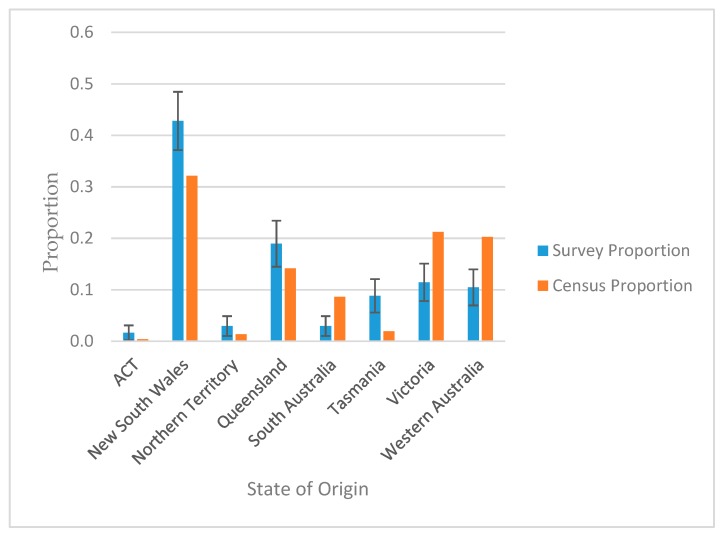
Representation of survey participants relative to general population, as reflected by the 2016 Australian census. Double standard error bars are shown, i.e., the bars represent twice the standard error above and below the predicted mean.

Participants represented all states and territories. Figure 1 shows the proportion of respondents by state, along with double standard error bars (i.e., the bars represent twice the standard error above the predicted mean; based on an assumed multinomial sampling distribution) and how the current survey data relate to the 2016 Australian census [32]. If the error bars do not overlap the census proportion, then the hypothesis that the survey is representative for that state can be rejected assuming an approximate normal distribution for the corresponding z-score test statistic (*p* < 0.05). On this basis, relative to the general population, the Australian Capital Territory, Northern Territory, New South Wales, Queensland and Tasmania were over-represented, Victoria and Western Australia were under-represented and South Australia was greatly underrepresented.

Among the respondents who completed the survey, 262 (85.6%) were female and 44 (14.4%) were male. The highest proportion of female participants was in the 46–60 age group and the highest proportion of males were over 60 years of age. Most respondents were over the age of 46 years (*n* = 213, 69.6%). Few respondents were under the age of 30 years (*n* = 38, 12.4%). Nine participants identified as aboriginal and/or Torres Strait islander (*n* = 306, 2.9%). Most respondents were born in Australia (*n* = 228, 74.8%) and had English as their first language (*n* = 306, 96.4%).

A significantly higher proportion of females nominated the motivation to help animals compared with males, and a significantly higher proportion of males nominated the motivation to conserve the environment compared with females. Other motivations where there were significant differences were securing a future job with animals, having the skills to rear animals, socializing and meeting people and filling a void in their lives. All of these were stronger motivations for females than for males (See Table 4).

The longest period that a participant was involved as a wildlife carer was 50 years (from 1968 until 2017), with four participants having been involved for over 45 years. The average duration of participation in the sector was approximately 11.4 years (*n* = 305). Approximately one third (32.8%) of the respondents had spent less than five years caring for wildlife (*n* = 100).

Respondents (*n* = 236) reported an approximate total time spent on training, mentoring and attending meetings, rescuing and/or rehabilitating fauna, record keeping, fundraising activities, travelling or community public relations activities in 2016–2017. The total of the average hours worked across respondents was 9994, at an average of 31.6 h·week^−1^. wildlife carer^−1^.

Respondents reported the total cost of animal food and associated equipment and infrastructure, travel and stationery, veterinary fees, training course fees, membership fees and insurance. For the year ending 30th June 2017, the total financial contribution by the respondents (*n* = 232) was AUD1,560,269 (mean of AUD5307 per carer; standard deviation of AUD10,574), with the maximum being AUD89,000.

**Figure 2:**
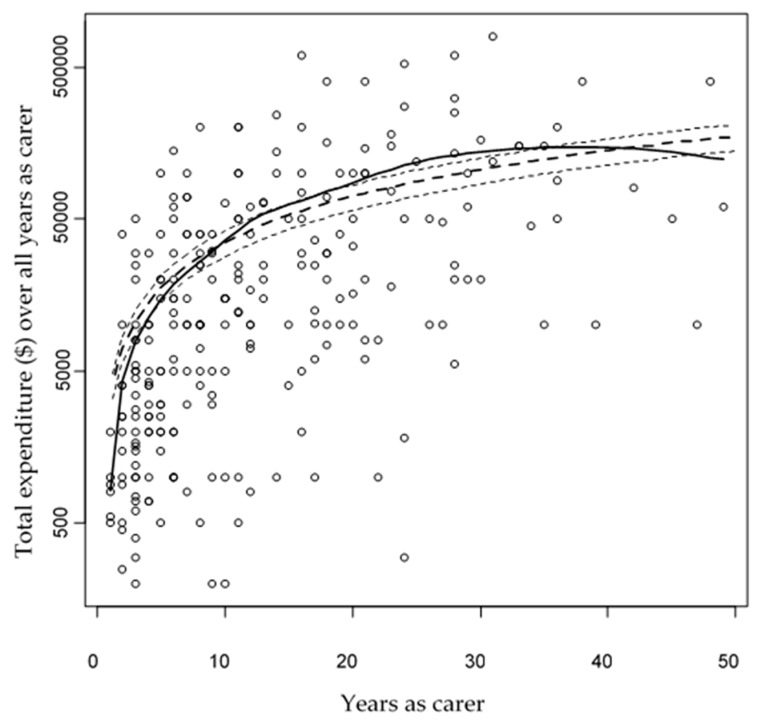
Total expenditure by wildlife carers during the time they have been caring for wildlife versus years as carer. The solid line is LOESS fit and dashed line is the generalized linear model (GLM) fit with double standard error bounds shown as thin dashed lines.

Respondents gave an approximate overall total for the personal funds they have spent on caring for animals during the time they have been a wildlife carer. This included any income they lost if they needed to take time off work (e.g., to take a joey to a veterinary clinic or nurse a sick joey). The total expenditure by the respondents (*n* = 293) was AUD13,607,915 (mean AUD46,443; standard deviation AUD98,098). Figure 2 shows the relationship between years as a carer and total expenditure (on the base 10 logarithmic scale) by wildlife carers during the time they have been caring for wildlife. The model with the lowest AIC and therefore the best model according to this criterion included the above terms with the exception of average hours, so this last term was dropped from the model. The fitted GAM explained 16.7% of the null deviance (i.e., in the case of a Gaussian response variable the null deviance is the residual variance for the simple mean model). The GLM predicts that the average annual expenditure per carer is AUD3513 (SE = 340).

**Figure 3:**
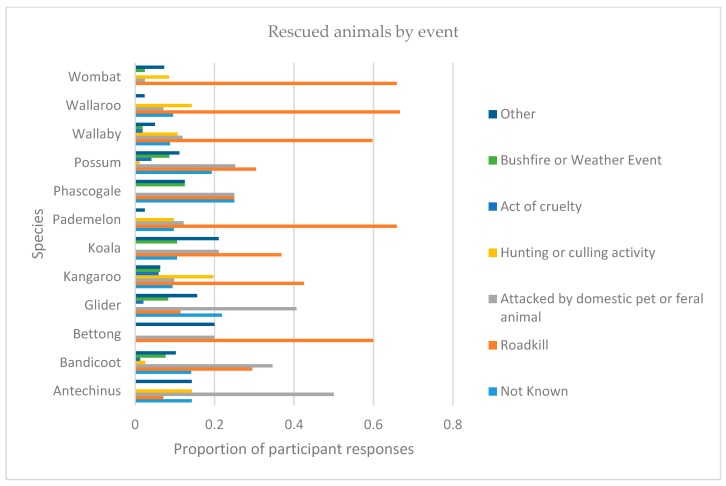
Animals by species and the reason for their rescue.

There are two major types of event that differentiate the rescue of smaller and larger marsupials. Having been attacked by domestic pet or feral animal is the main reason that *Antechinus*, bandicoots and gliders (i.e., smaller species) require rescue, while roadkill is the main reason for the rescue of kangaroos, koalas, pademelons, possums, wallabies, wallaroos and wombats (i.e., relatively larger animals). Proportionately, roadkill and attack by domestic or feral animal accounted for most of the injured or orphan animals taken-in by carers (Figure 3).

A total of 4608 joeys were raised by the respondents (*n* = 305) in the 12-month reflective reporting period 2016–2017, with an average of 15 animals·carer^−1^·year^−1^. The species raised were predominantly possums (*Trichosurus* sp.; *n* = 1681, 36.48%), kangaroos (*Macropus* sp.; *n* = 1009, 21.89%), wallabies (*Macropus* sp.; *n* = 614, 13.31%) and wombats (*Vombatidae* sp.; *n* = 327, 7.10%). Those four species accounted for nearly 80% of all animals reared. Gliders (*Petauridae* sp.; *n* = 290, 6.29%), bandicoots (*Peramelemorphia* sp.; *n* = 205, 4.44%), wallaroos (*Macropus robustus*; *n* = 127, 2.75%) and pademelons (*Thylogale* sp.; *n* = 124, 2.69%) were the four next represented, totaling 16%. Least represented were *Antechinus* (*Antechinus* sp.; *n* = 88, 1.9%), koalas (*Phascolarctos cinereus*; *n* = 72, 1.56%), phascogales (*Phascogale tapoatafa*; *n* = 23, 0.4%) and bettongs (*Bettongia* sp.; *n* = 22, 0.47%), followed by Tasmanian devils (*Sarcophilus harrisii*; *n* = 7, 0.15%), quolls (*Dasyurus* sp.; *n* = 7, 0.15%), potoroos (*Potorous* sp.; *n* = 6, 0.13%), dibblers (*Parantechinus apicalis*; *n* = 3, 0.06%), dunnarts (*Sminthopsis* sp.; *n* = 2, 0.04%) and a quokka (*Setonix brachyurus*); *n* = 1, 0.02%). Seven Australian native species were not represented: bilby (*Macrotis lagotis*), kowari (*Dasyuroides byrnei*), marsupial mole (*Notoryctes typhlops*), mulgara (*Dasycercus* sp.), ningaui (*NingauI ridei*), numbat (*Myrmecobius fasciatus*) and planigale (*Planigale maculata*).

A total of 722 joeys died whilst in care of the respondents (*n* = 276) in the 12-month reflective reporting period, with an average of 2.6 animals·carer^−1^·year^−1^. When compared with the average number of animals reared and released, i.e., 15 animals·carer^−1^·year^−1^, this represents a success rate of over 85%. Wildlife carers were asked about how they implemented pre-release training or conditioning of animals. Over 70% of wildlife carers reported that they taught the rescued animals two lessons: learning how to find their own food (forage; *n* = 238, 82.6%) and how to interact with their conspecifics (*n* = 206, 71.5%). Four other lessons were taught by approximately half the carers: finding water (*n* = 168, 58.3%); avoiding pet animals (*n* = 165, 57.3%); avoiding humans (154, 53.5%); and, seeking shelter or digging a den (*n* = 143, 49.7%). Few carers claimed that they trained the animals to avoid or evade predator species (*n* = 84, 29.2%) or to avoid motor vehicles (*n* = 40, 13.9%), and 65% (*n* = 188) had received no training in animal behavior modification techniques.

**Figure 4:**
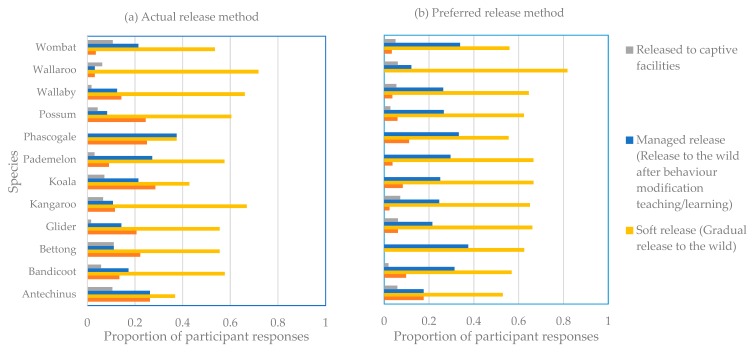
Required and preferred methods used to return rehabilitated wildlife to the wild. (**a**) The actual method required by legislation and (**b**) the method preferred by wildlife carers.

For all animals reported by the current respondents, the primary method used to release them was a soft release while the second most popular method used was a hard release (Figure 4a). Significantly, for releasing wombats, hard releases were the least used, with managed releases being the second most popular method. Given a choice to select a method of release for all animals, a managed release was selected as the second most popular method, rather than a hard release (Figure 4b).

**Figure 5:**
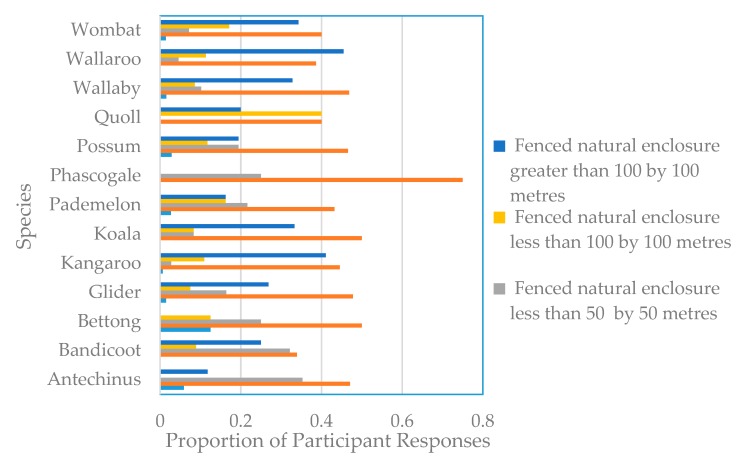
Preferred place of temporarily housing rehabilitated animals prior to release.

Rehabilitated animals may require extra time in captivity for various reasons including behavioral and health assessment, integration with conspecifics, inappropriate weather or season for release and availability of suitable habitat. However, depending on the species in question, wildlife carers preferred to retain the animals on their property and so use facilities that were relevant to the physical size of the animal and its behavioral needs of space. Least favored was to use a zoo or wildlife park to house the animals (see Figure 5).

In deciding the preferred option for animals deemed unsuitable for release, two-thirds of the wildlife carers (*n* = 202, 67%) preferred the options of the animal being kept on their private property or at a secure enclosure, rather than euthanasia. However, some respondents also indicated that they would prefer an animal to be euthanized (*n* = 51, 17%) rather than be retained in a zoo or wildlife park (*n* = 45, 15%).

Information was sought on methods used to identify animals in rehabilitation and whether any feedback was received after release. An estimated 3900 animals were reported to have been released by the respondents (*n* = 289). Upon release, only 4.7% of the animals released were identifiable in an objective manner; by microchip (2.2%), ear tag (1.5%) or tracking collar (1%), i.e., one that did not rely on the knowledge or expertise of an individual carer. Respondents reported that they were able to identify individual animals by visual appearance (32%), by individual behavior traits (20.2%) and other unspecified means (3.7%). Thus, 39.4% of released animals were not identifiable.

Feedback on the fate of animals that had been released was species-dependent, with the smaller species (*Antechinus*, bandicoot, glider, phascogale and quoll) generating the least amount of feedback. Over 50% of the respondents received feedback about the eight larger species, which aligns with the reported 57% of animals being identifiable (as shown in Table 5). However, even if visual (32%) and behavioral observations (20.2%) are accepted as reliable, there still remain 39.4% of animals for which there is no record of outcome after release.

Approximately half of the carers (*n* = 155, 49%) agreed or strongly agreed that the current methods of release were the best that could be achieved. However, given that slightly over half of the respondents were either being unsure (*n* = 92, 29%), disagreed or strongly disagreed (*n* = 70, 22%), this is an area that merits attention.

The mental health of wildlife carers was examined by the type of loss they had experienced. The loss of an animal in care through death was the most frequently reported event that caused distress in the previous 12 months (*n* = 212, 72.4%).

An assessment of the grief experienced by wildlife carers in the year 2017–2018 was conducted with 30 of the 316 total respondents declining to answer questions on loss and grief, and 12 did not complete all the questions in this section. The collated data on participants’ responses to 16 grief-related questions revealed that six respondents reported having experienced no grief (*n* = 274, 2.2%), 119 reported minimum grief (*n* = 274, 43.4%), 72 reported mild grief (*n* = 274, 26.3%), 30 reported moderate grief (*n* = 274, 10.9%) and 47 reported severe grief (*n* = 274, 17.2%).

**Figure 6:**
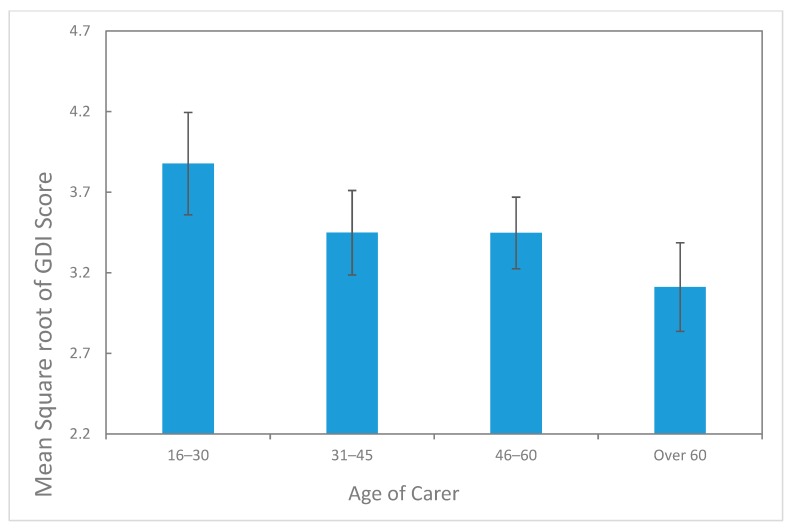
Predictions of grief diagnostic instrument (GDI) score on the square root scale versus Age Category of Carer for Years as Carer fixed at 5 and Number of Joeys Died set to the average of 2.6 and log of costs set to 9 (i.e., AUD8103). SE bars are shown.

**Figure 7:**
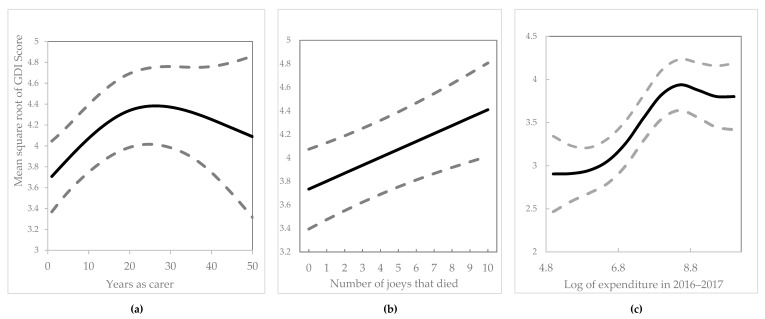
Predictions of GDI score on the square root scale for age category of carer fixed at 16–30 versus (**a**) years as carer for the number of joeys died set to the average of 2.4 and log of costs set to 9 (i.e., AUD8103), (**b**) number of joeys died for years as carer set to 5 and log of costs set to 9 and (**c**) log of the financial cost in the previous year for years as carer set to 5, and number of joeys died set to the average of 2.4. Standard error bounds are shown as dashed lines.

For the 274 respondents who completed all 16 questions, Cronbach’s alpha was calculated using the R-library ltm [33] at 0.94 with 95% bootstrap confidence limits ranging from 0.93 to 0.95. This demonstrates a very high degree of reliability of the GDI scores. The R-library mgcv was used to fit the GAM and obtain predictions, and for residual histograms and qq-plots [31]. The outputs from the fitted GAM, presented in Figure 6 and Figure 7, show that the 60 years and over age category had the lowest average GDI score, after accounting for the other significant predictor variables, while the 16‒30 years age category had the highest average, with the intermediate age categories showing intermediate levels of average GDI score. The difference between the 16–30 years and 60 years plus age category GAM parameter estimates was highly significant (*p* = 0.025) as obtained from the corresponding t-statistic, while for the other two age categories parameter estimates were not significantly different from the estimate for the 16‒30 years age category (Figure 6). As years of caring increased up to a peak at around 20 years, the average GDI score also increased, after accounting for the other significant predictor variables, and plateaued afterwards (Figure 7a).

Average GDI score increased linearly with the number of joeys that had died in care in the previous year, after accounting for the other significant predictor variables (Figure 7b).

Average GDI score increased as financial cost in the previous year increased, but plateaued when cost reached AUD$8000 for the 2016–2017 year, after accounting for the other significant predictor variables (Figure 7c). Financial cost was the most significant of the predictor variables, with its spline term highly significant (*p* < 0.005), and the precision of the estimated curve was much higher than the predicted trends for the other two continuous predictor variables.

Of the respondents who responded to questions about the support they received and their own wellbeing, 95 (32.1%) stated that they felt supported enough as a wildlife carer at the organizational/government level to achieve optimal outcomes for the animals they reared, 107 (36.0%) stated that they felt supported enough for their own wellbeing and 221 (74.9%) stated that immediate support was available should they become ill or injured.

There were 152 responses (*n* = 316, 48.1%) to the open question inviting details on the role carers play in rescuing, rehabilitating and releasing native marsupial wildlife. Fourteen different types of concerns were expressed by more than one individual respondent. The greatest concerns expressed were lack of government support (*n* = 47), financial pressures (*n* = 32), mental health problems (*n* = 25) and lack of public care (*n* = 17). Other concerns were lack of wildlife carers and interpersonal issues (*n* = 12), the release of animals to the wild with no ongoing monitoring (*n* = 11), destruction of habitat and lack of knowledge about wildlife rescue among the public (*n* = 10), abuse by the public (*n* = 8), physical health problems (*n* = 7) and burnout (*n* = 6). Lack of knowledge (*n* = 5) and lack of veterinary input (*n* = 3) were the least considerations.

A more detailed analysis of the three main concerns (lack of government support, financial pressures and mental health problems) suggests that wildlife carers felt they have a general lack of central government support and that their work is not valued or even understood by government agencies. The issues detailed were: general lack of central government support (*n* = 22); lack of understanding and work not valued (*n* = 9); lack of government financial support (*n* = 5); poor regulation of wildlife carers (*n* = 5); lack of agency support; e.g., Parks and Wildlife (*n* = 4) and culling of wildlife by government agencies (*n* = 2). They also considered that some of the public have no regard for animal welfare and that some do not understand or appreciate their work. The issues detailed were: a no care attitude of the general public (*n* = 8); the need to educate the public about the work wildlife carers undertake (*n* = 6); lack of recognition (*n* = 5); lack of general understanding (*n* = 4); lack of public support (*n* = 2) and intolerance from the public (*n* = 2). A specific example was a lack of understanding that wildlife carers are volunteers who contribute their time, are self-funded and do not receive any financial remuneration for the service they provide. Some members of the public expect them to attend at a moment’s notice and clear up the mess of a roadkill victim. Nearly two-thirds of the respondents who cited mental health issues (*n* = 16) as a problem had feelings of depression, disillusionment, grief, isolation, emotional issues or mental stress. The issues detailed were: emotional issues and mental stress (*n* = 9); feelings of depression, disillusionment, isolation and grief (*n* = 7); feelings of guilt and failure at having to refuse to take in additional animals (*n* = 4); managing non-core stressors, e.g., administration tasks (*n* = 3) and not knowing what happens to animals after they are released (*n* = 2).

## 4. Discussion

Wildlife carers from all states and territories of Australia were represented in this survey, with adult respondents of all ages and both genders reporting on their care for injured and orphaned native wildlife. South Australia was significantly under-represented perhaps because a large South Australian wildlife carer organization stated it was unable to participate in this research project due to their members’ high workload and so declining to distribute the survey to its carers. Therefore, caution is warranted as this may have hindered the power of statistical analysis when assessing whether stress, caused by a high workload, was a factor in possible compassion fatigue and carer burnout.

As the number of animals needing rescue and rehabilitation increases with external threats, the workload for the wildlife carers is likely to increase, exacerbating workload-related stress. Of particular concern for 25% of the respondents is the lack of emergency support. There are severe consequences for those animals requiring feeding at regular intervals, should their carers become incapacitated for any reason. The perceived lack of government or agency support at 68% and 64%, respectively, is also of concern. A similar dissatisfaction rate has been reported from other studies both nationally and internationally [2,15,34,35,36].

Among the reported motivations for becoming a carer, animal welfare, conservation of the environment and contributing to the community were the top three reasons for participating in wildlife rehabilitation given in a survey of wildlife rehabilitators in New South Wales [2] and the top two in a survey of wildlife carers in Queensland, New South Wales, Victoria and South Australia [7]. These motivations reflect a one welfare approach [37] contributing to the common good of animals, humans and the environment. From an international perspective, similar results were presented in studies in other countries [26,34,36,38]. However, in the current survey, there were significant gender differences, in particular for the two dominant motivations across all respondents of “having an affinity with animals” and “conserving the environment”. The ratio of female:male wildlife carers was 85.6:14.4 in the current study; similar to those obtained in other surveys of wildlife carers, with 79:21 in NSW [39], 77:23 in New Zealand [24] and 87:13 for Australia [15]. However, when describing wildlife carers, Tribe and Brown [15] reported that in 1997 the mean age in Victoria was 44 years and in New York 42 years. These figures contrast with the current study where, in Australia in 2018, 70% of wildlife carers were over the age of 46 years and 25% were over the age of 60 years, with a mean age of 49.6 years, indicating an increasingly aging demographic. In a recent survey of wildlife carers in NSW [2], the average duration of participation in the wildlife sector was nine years, with the longest period serving as a carer being 65 years. Furthermore, that study reported flux in the membership of organizations that ranged from 25% to 60%. These average durations, coupled with 70% of wildlife carers being over the age of 46 in the current study, suggest that over the next ten years there will be a need for large-scale recruitment even to retain the current number of wildlife carers.

In many ways, nursing can be considered a profession that is similar to wildlife caring. Roche et al. [40] reported that, in an Australian hospital setting, the annual turnover of nurses with more than 5 years’ experience was 20%. International comparison of turnover rates reveals similar figures. A survey of the turnover of nurses in a major medical centre in America [41] highlighted that staff turnover was greatest in the first year of recruitment at 39%, falling to 17% by the end of the fifth year and then remaining at 17% for the following years. If applied to the 32.8% of wildlife carers in the current cohort with less than five years’ experience, such annual turnover rates emphasize the need for an increase in recruitment. One significant area where recruitment might be boosted is in the older male population where, as revealed in our results, 57% of male wildlife carers are over 60 years old. It may be that, given the opportunities that financial stability, spare time and reasonable health can provide at retirement age, males could be encouraged to take up an animal caring role as a new interest. It is worth noting that conserving the environment was cited as the main motivation for becoming a wildlife carer among male respondents in the current study.

Internal threats to the people who volunteer to take on the responsibility of caring for injured and orphaned wildlife are financial, physical and mental. From the financial perspective, the average annual expenditure of AUD5307 incurred by wildlife carers surveyed in this study contrasts with figures quoted in a report on wildlife volunteers in New South Wales in 2017 [2], in which primary animal carers estimated an average annual expenditure of AUD4000. This difference in expenditure probably reflects the NSW study’s inclusion of all wildlife carers, whereas the current study focused only on those caring for marsupials. Birds, reptiles and bats account for more than half of the animals rescued, generally require less time in rehabilitation and accordingly attract lower feed and infrastructure costs [2,3,25]. Some wildlife carers contribute up to AUD800,000 over a lifetime of caring for wildlife, considerably more than the 2015 average individual Australian superannuation fund balance at retirement (AUD292,500 for men and AUD138,150 for women [42]). These monetary values demonstrate the high financial burden placed on anyone volunteering to be a wildlife carer. In all jurisdictions, regulations state that wildlife carers themselves must meet all the costs involved in rescue and rehabilitation [4].

The physical effort required of wildlife carers is considerable. The Australian Bureau of Statistics gives an average of 2.5 h·week^−1^ as the contribution of general volunteering in 2014 [43]. Average actual hours worked per week by Australians in all jobs generally decreased over the 32 years from 1978 until 2010, from approximately 35.5 h in early 1978 to approximately 33 h in 2010 [44]. A significant number of wildlife carers (*n* = 141, 45%) devote more hours than these average 33 working hours and over 90% of wildlife carers volunteer more than 2.5 h·week^−1^. Some respondents invest up to 100 h·week^−1^ caring for animals, much more time than if they were in full-time paid employment. The current survey did not reveal how many carers were in full or part-time paid employment. However, the physical burden of working such long hours is considerable and likely involves sleep deprivation.

The cognitive challenges of rescuing and rehabilitating animals need clarification. Wildlife carers are obliged to constantly update their knowledge base and navigate complex legislation and also manage detailed record-keeping [4]. They are mandated in the way that they have to keep and release animals, although these may not correspond with their knowledge, experience and preferences. Slightly more than half of the current respondents did not believe that the methods currently used to release animals are optimal and, given a choice, they would alter the method of release, with behavioral modification of the animals being granted a much higher priority than is currently the case, while acknowledging that they would need professional training to accomplish this.

From the perspective of an animal, motor vehicles could be regarded as a reasonably novel threat to wildlife. Like historic threats, such as predators, they kill or injure, strike randomly, move at high speed and with little warning. This motor vehicle threat must appear alongside the other predatory threats to which, as hand-reared orphans, these animals are naïve. In a survey of rehabilitation practices in 2010, Guy and Banks [7] noted that antipredator training was conducted by only 20% of respondents. In contrast to their report, the current study reveals an increase to 29.2% in anti-predator training by wildlife carers over the ensuing seven years. However, even at this percentage, there remains a significant gap in this aspect of behavioral training. This could indicate that carers realize there is a shortfall in professional training in animal behavior modification techniques but, given training, would apply these techniques to animals prior to releasing them. These methods could be applied to considerable advantage with those animals that may have inadvertently been desensitized to humans, vehicles and companion animals through habituation during the rehabilitation process.

That it is possible to successfully introduce aversion behavior modification techniques has been demonstrated in the reintroduction of quolls [45], prairie dogs [46] and possibly greater bilbies [47]. Without antipredator training prior to release, the attrition rate among prey species can be high, as happened with eastern quolls recently reintroduced to mainland Australia; 70% were dead within three months of being released [48]. It may be that the highly successful release of 54 hand-reared wombats monitored over a period of eight years and released using seven release pens, as a soft/managed release protocol, set a precedent for the release of wombats and perhaps other species [49]. Although reintroduction biology emerged as a new science in Australia and New Zealand only some 20 years ago [50], the understanding of the process of reintroduction has advanced [51]. This is an area where research and practical application could address a persistent void in behavior modification training for wildlife carers.

From the time they rescue or accept an animal for rehabilitation, wildlife carers face various emotional challenges. They commonly confront the necessity to have rescued animals euthanized or witnessing them die during the rehabilitation process. Approximately 86% of wildlife carers in the current study were female and they reported that the primary motivation for becoming a wildlife carer was having an affinity with animals, whereas for males this was the secondary motivation, the first being conservation of the environment. Previous research indicates that having an affinity with animals makes the euthanasia or death of animals a significantly distressing event [52,53,54]. Thus, female wildlife carers who cited having an affinity with animals as their main motivation for becoming a wildlife carer seem especially vulnerable to distress that may lead to feelings of anger, sadness, guilt, fear, depression and helplessness [54,55,56,57,58] when confronted with the possibility of having an animal euthanized.

In the current study, euthanasia was the third preferred option for an animal deemed unsuitable for release into the wild, which contrasts with the findings of a survey of rehabilitation practices in the eastern states of Australia, conducted in 2012 [7], where euthanasia was the most preferred option. This presents a dilemma for carers, most jurisdictions mandate euthanasia for animals deemed unsuitable for release [4]. In Queensland and the Australian Capital Territory, there are mechanisms whereby the animal may be placed in a wildlife park or zoo. However, this option was the least favored option among the current respondents, who preferred the animals to be euthanized (*n* = 51, 17%) rather than be retained in a zoo or wildlife park (*n* = 45, 15%). This appears to be a surprising finding, given that these are institutions where expertise is likely available to ensure the animal will have a life worth living, if remaining in captivity.

An animal may require temporary housing before release, for reasons that may include integrating a gregarious animal with conspecifics, undertaking a behavior modification program, quarantining for health reasons, recovery from injury or so that an assessment can be made as to its suitability to be returned to the wild. Again, it is interesting to note that, of all the options for temporary housing, the places where professional input for all the above factors would be available, i.e., a zoo or wildlife park, are the least favored by carers (Figure 5).

CF is a condition resulting from a decline in compassion among those caring for others, whether human or animal. It was defined by Figley as the “cost of caring” [17]. This may well be the outcome for wildlife carers, with 77 of the current respondents (*n* = 274, 25%) reporting moderate to severe grief at constantly being faced with seeing dead animals, euthanizing animals or having them die during rehabilitation. Further confirmation of this outcome came with evidence that the grief they experienced when animals died in their care increased in direct correlation with the mortality rate. Given that a general grief instrument was used for the current study, caution is required when interpreting the grief results. The grief measured may not be entirely due to distressing events during the process of caring for wildlife, and may include other life events. However, the GDI does enable the variety of loss variables to be taken into account. Wildlife carers in the current study also mentioned the culling of wildlife by government authorities as a concern, which highlights another dilemma they face. In one context, wallabies and kangaroos are seen as pest species to be culled [59,60,61], whereas in another context wildlife carers work to save members of these species, only to acknowledge that these animals would possibly be killed after release; a conflict that may be philosophically disquieting for the carers, and compound stress.

Respondents to the current survey also reported on the predicament that, after spending up to two years rearing an animal, they have to release it into the wild with no reliable method of identification, so that they are unlikely to ever know what happened to the animal after release. Given that conservation of the environment was stated as the highest motivation for male respondents and the second highest for female respondents, it is difficult to believe that carers’ motivations are being met satisfactorily, especially given that the current lack of reliable identification makes it unfeasible to monitor what happens to animals post-release.

Respondent wildlife carers reported bullying, intimidation and a lack of concern and compassion from peers, wildlife organizations and the general public. Similarly, Haering et al. [2] reported ‘infighting and bullying’ in a survey of wildlife carers in NSW and Carleton [25] stated that bullying was experienced by 39% of participants in an international survey of wildlife carers.

The effect of all the above factors, in combination, on the mental health of the wildlife carers merits consideration. The current analyses reveal that, regardless of the number of hours worked, the lower the age of wildlife carers, the longer the length of service in wildlife caring, the greater the financial input and the greater the number of joeys that died during rehabilitation, the more susceptible the respondents were to a GDI score ranging from moderate to severe. Clark et al. state that a GDI score of 18 or above for a patient in a general medical practice setting indicates a need for further assessment [28]. In a review of roadkill rescue that includes a section on mental health, Englefield et al. [3] posit that wildlife carers could be susceptible to 17 of the 20 established types of grief. The current study reveals 77 respondents scoring a GDI of 18 or above (*n* = 274, 28%), indicating moderate to severe grief. This suggests the need for wildlife carers to have access to counseling from a professional mental health practitioner as well as to programs that build resilience. Unfortunately, two-thirds of respondents reported that their personal mental health was not supported.

Caregiver burnout is a manifestation of physical, emotional and mental exhaustion. Caregivers who are burned out may experience fatigue, stress, anxiety and depression [62,63]. Wildlife carers who are physically challenged by experiencing sleep deprivation through 4-hourly feeding of joeys and working over 40 h a week (26%), who are emotionally challenged by animals dying or being euthanized (92%), experiencing bullying, isolation and mental challenges (16.5%), who are under financial pressures (38%) and experiencing moderate to severe grief (28%) are likely to suffer caregiver burnout, given that they are subject to a combination of physical, emotional and mental stress. This could result in wildlife carers having to stop rehabilitating animals. Unless replacements can be recruited and are trained to be resilient and receive appropriate support, this will lead to an exponential spiraling effect where the workload will increase for the remaining wildlife carers, leading to more of them burning out and more animals going without care. Within this emerging domain of understanding compassion fatigue, burnout, resilience building, psychosocial and organizational support and PTSD, there is much to be studied providing an avenue for future research.

## 5. Conclusions

Australian wildlife rescue, rehabilitation and release all rely heavily on the voluntary effort of wildlife carers. Carers provide a valuable service to the community, through organizations and as individuals, but many feel unappreciated, undervalued and lacking in support. The work they undertake is demanding physically, financially, emotionally and mentally. External and internal threats to the Australian wildlife rescue sector are increasing. It is likely that, in the immediate future, unless changes are made to reduce roadkill and to increase the recruitment of carers with financial, emotional and mental support given to them, viable animals will need to be euthanized in larger numbers, more roadkill victims will remain unattended and animal and wildlife carer wellbeing will be compromised.

## Figures and Tables

**Table 1:** Questions asked of wildlife carers.

	Questions Relevant to the Year July 1st 2016 to June 30th 2017
1	In which year did you become a volunteer wildlife carer
2	Which of the following Australian native marsupial joeys; rescued as orphans, have you hand-reared
3	How did the joeys you hand-reared become orphans
4	Give an approximate figure for the total number of rescued joeys that died whilst you were caring for them
5	What teaching did the animals receive before they were released
6	What was the method used to release any animals
7	Were the animals identifiable
8	Did you receive any feedback indicating what happened to the animals after they were released?
9	Have you received any formal instruction or training in animal behavior modification techniques?
10	Which method would you prefer to use to release the animals you reared
11	Which method would you prefer to see used as a temporary home
12	What should happen to an animal that cannot be released?
13	Are the methods of release that are currently being employed optimal for the welfare of the animals
14	Approximately how many hours per week on average did you spend doing volunteer work as a wildlife carer?
15	What was your approximate personal financial contribution?
16	During the time you have been a wildlife carer give an approximate overall total for the personal money you have spent on caring for animals?

**Table 2:** Distressing events that had impacted wildlife carers.

	Questions Concerning Distressing Events Wildlife Carers Had Experienced
1	Receiving adverse comments about the way you care for animals
2	Rescuing pouch-young or an injured joey from a dead female
3	The death or serious illness of an animal in your care
4	Releasing animals to the wild and not knowing whether they survived or had a life worth living
5	Had an animal taken by authorities, hard released to the wild and which died shortly afterwards
6	Financial hardship as a result of the cost of caring for animals
7	Health-related loss
8	Loss of freedom through being a carer
9	Other

**Table 3:** Losses experienced by the wildlife carers and the effect of these losses on their thoughts and behavior.

	Questions about the Effect of Experiencing the Losses Experienced by the Wildlife Carers
1	Have thoughts of the losses made it difficult for you to concentrate, remember things or make decisions
2	Have you experienced images of the losses surrounding the event
3	Have you found yourself longing for what has been or will be lost
4	Have reminders of the loss caused you to feel longing for what has been or will be lost
5	Have thoughts or reminders of the loss caused you to feel guilt
6	Have thoughts or reminders of what has been or will be lost caused you to feel sick or ill in any way
7	Have thoughts of the loss come into your mind whether you wish it or not
8	Have you felt distress by the reality of the loss
9	Have thoughts or reminders of the loss caused you to feel dread of the future
10	Have thoughts of your loss caused you to be more irritable with others
11	Overall, how much have thoughts and feelings about your loss or losses distressed you
12	Have other animals, people or familiar objects reminded you of the loss
13	Have thoughts or reminders of the loss caused your emotions to feel numb
14	Have you found yourself imagining that the loss did not or will not occur
15	Have reminders of the loss caused you to feel sadness
16	Have thoughts or reminders of the loss caused you to feel anger?

**Table 4:** Motivations that prompted respondents into becoming a wildlife carer. The respondents could select as many of the motivational reasons as they felt applied to them.

Motivation	Number of Respondents			Percentage of Responses
	Female	Male	Total	
I had an affinity with animals and a desire to help them	209	29	238	88.5
To help conserve the environment	155	31	186	69.1
To contribute to my community	93	14	107	39.8
I had the skills to rear animals	93	11	104	38.7
I related better with animals than humans	70	9	79	29.4
To learn something new	62	7	69	25.7
To handle animals	63	10	73	27.1
To challenge myself	57	4	61	22.7
I believed that I had a special gift in relating to animals	56	8	64	23.8
To socialise and meet people	21	2	23	8.6
To do something different	23	3	26	9.7
To fill a void in my life	24	1	25	9.3
To help me get a future job with animals	12	1	13	4.8

**Table 5:** Feedback that was received by wildlife carers relevant to the species they released to the wild.

	Total Animals Released	Feedback Received		SE
		Yes	No	
*Antechinus*	15	2	13	0.18
Bandicoot	46	17	29	0.14
Glider	58	3	4	0.13
Kangaroo	117	31	27	0.09
Koala	10	7	3	0.29
Pademelon	27	17	10	0.19
Phascogale	8	0	8	0.00
Possum	189	102	87	0.07
Quoll	5	0	5	0.00
Wallaby	103	66	37	0.09
Wallaroo	32	23	9	0.16
Wombat	57	37	20	0.13
